# The "Break-Up" of the Poor-Law

**Published:** 1909-05-15

**Authors:** Beatrice Webb, Sidney Webb

**Affiliations:** 41 Grosvenor Road, Westminster; 41 Grosvenor Road, Westminster


*To the Editor of* The Hospital.

### THE "BREAK-UP" OF THE POOR-LAW.

*To the Editor of* The Hospital.

Sir,—The rival Reports of the Poor Law Commission have now been before the public for nearly three months. The evils laid bare by both those Reports—the far-reaching demoralisation of character, the failure to cope with the destitution of literally hundreds of thousands of children, the lack of medical treatment of incipient disease, the degradation and suffering involved in the general mixed workhouse, with its mixture of good and bad, sane and feeble-minded, old and young, the waste of public money implied by the spending of seventy millions a year by overlapping authorities, in duplicated services—are still going on. The emphatic condemnation of the "principles" of the existing Poor Law, in which both Reports concur, cannot fail to make the local administration even more confused and more divergent than it has been. It is time that those who believe in the "Break-up" of the Poor Law, the abolition of the workhouse, a systematic attempt to prevent unemployment, and the wisest possible provision for each class of persons needing public assistance should draw together and organise their forces.

It has been decided to form a National Committee to promote the break-up of the Poor Law on the lines of the Minority Report. What is desired is the co-operation of those who are willing to help in any one of the following ways: (*a*) By lending their names and local influence; (*b*) by writing, lecturing, or personally helping in organising and office work; (*c*) by contributing money. It is intended that subscription should be entirely optional; but funds must be raised for printing, postage, meetings, and the travelling expenses of lecturers, and those able to contribute money are requested to do so. The Committee will need £1,000 for its first year's work. One lady has sent me £50 for a start.

A meeting will be held shortly, to which all who have sent in their names will be invited, and at which an Executive Committee and officers will be appointed. In the meantime I am acting as Secretary, and I would ask all in sympathy to write to me. I am, etc.,

Beatrice Webb (Mrs. Sidney Webb).

41 Grosvenor Road, Westminster, May 7, 1909.

